# Effect of Protonation on Optical and Electrochemical Properties of Thiophene–Phenylene-Based Schiff Bases with Alkoxy Side Groups

**DOI:** 10.1021/acs.jpcb.1c05390

**Published:** 2021-07-27

**Authors:** Paweł Nitschke, Bożena Jarząbek, Andra-Elena Bejan, Mariana-Dana Damaceanu

**Affiliations:** † Centre of Polymer and Carbon Materials, Polish Academy of Sciences, 34 M. Curie-Skłodowska Str., Zabrze 41-819, Poland; ‡ Electroactive Polymers and Plasmochemistry Laboratory, “Petru Poni” Institute of Macromolecular Chemistry, Aleea Grigore Ghica Vodă nr. 41A, Iaşi 700487, Romania

## Abstract

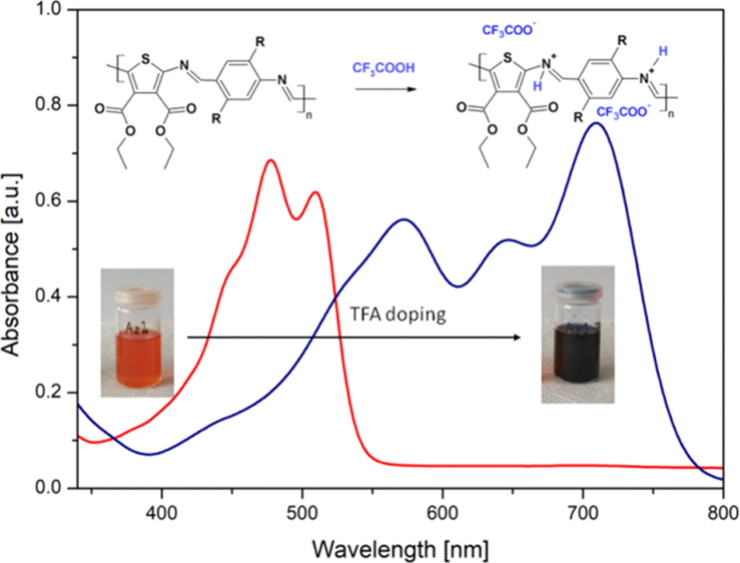

Three polyazomethines and their corresponding model compounds were protonated with trifluoroacetic acid, and its effect on their optical (UV–vis absorption and photoluminescence) properties and electrochemical behavior has been studied, in the context of the presence and elongation of alkoxy side groups. Moreover, the effect of environment dielectric constants (i.e., polarity of the solvent) was considered on the doping process. It has been proven that the presence of alkoxy side groups is necessary for protonation to occur, while unsubstituted compounds undergo hydrolysis to constitutive units. Acid doping of imines consisting of alkoxy side chains has resulted in a distinct bathochromic shift (>200 nm) of the low-energy absorption band. Even the length of alkyl chains has not affected the position of shifted bands; it has been observed that azomethines with smaller, methoxy side groups undergo the protonation process much faster than their octyloxy-substituted analogues, due to the absence of steric hindrance. The electrochemical studies of these alkoxy-substituted imines have indicated a better p-type behavior after protonation induced by the capability of the protonated form to easily oxidize in acetonitrile and to generate the native molecules. The environmental polarity has also had impact on the doping process, which took place only in low-polar media.

## 1 Introduction

Conjugated compounds have drawn much attention in the last decades, owing to their interesting properties, well suited for application as organic semiconductors in many optoelectronic systems, such as organic photovoltaics,^1,2^ organic light-emitting diodes,^3,4^ or field effect transistors.^5^

One group of these materials is represented by azomethines and polyazomethines (PAzs) (also known as imines or Schiff bases), consisting of an imine bond (−CH=N), which has proved to have an isoelectronic character with a vinylene bond.^6^ Such materials are much easier to synthesize, in contrary to carbon–carbon or vinylene-coupled compounds, which usually require stringent reaction conditions and rather extensive purification processes.^7^ Azomethines (PAzs) are products of condensation (polycondensation) reaction between amines and aldehydes. Such reaction may proceed under mild conditions, using inorganic or organic catalysts^8^ or even without using any, as previously shown.^9,10^ These types of materials were extensively studied due to their interesting thermal,^11^ electrical,^12^ and optical^13−15^ properties and also due to the possible activity in some optoelectronic systems, such as bulk heterojunction photovoltaic cells,^16−19^ perovskite solar cells,^20−22^ organic light-emitting devices,^23,24^ or electrochemical systems.^25,26^

Generally, doping of semiconductors is a well-established way to enhance their optical and electrical properties. For conjugated polymers, it is mostly achieved using chemical dopants, such as iodine (I_2_) or FeCl_3_.^27^ Halogen doping is also one of the most often used methods in the case of PAz thin films.^28,29^ Due to the presence of a lone electron pair connected with a nitrogen atom of the imine bonds, azomethines and PAzs may be also doped using either inorganic or organic acids.^30−32^ Such a process may lower the glass transition temperature of the doped material,^33^ improve its solubility,^33^ and increase its conductivity.^33^ Moreover, through the change in protonated molecule planarity,^33^ the optical^34,35^ and electrochemical^36^ properties are being modified. Protonation of the imine bond may also lead to a major increase in photoluminescence (PL) intensity,^34,37−39^ probably due to the deactivation of photoinduced electron transfer, responsible for the PL quenching in imine compounds.^40^ The importance of this phenomenon is significant, since it may enhance the power conversion efficiency of organic solar systems, consisting of such protonated PAzs.^41,42^ Despite many papers reporting this process, the effect of the chemical structure on the photochemical response of the azomethine compounds upon protonation is still not sufficiently studied.

This paper aims at exploring and discussing the influence of the chemical structure (i.e., alkoxy side group substitution and its length) on the protonation process of some PAzs and their model compounds (Az), using various molar ratios of trifluoroacetic acid (TFA). This study is focused on spectroscopic (optical absorption and PL) and electrochemical investigations of the TFA-protonated thiophene–phenylene-based Schiff bases with alkoxy side groups. Results presented herein are compared with the properties of non-protonated counterparts, which were already described in our earlier studies.^9,19^

The TFA doping process was monitored by ^1^H-NMR spectroscopy, to ensure that the chemical structure has not changed. The photochemical response was subsequently investigated by the UV–vis absorption and PL spectroscopic measurements, performed for solutions of different polarities. Additionally, the influence of the chemical doping process on electrochemical behavior was also observed using the cyclic voltammetry (CV) method. The comparison of spectra registered before and after protonation of the studied compounds has provided new information regarding the doping process of unsubstituted and alkoxy-substituted azomethine compounds, with a particular emphasis on the effect of solvent polarity on the optical properties. Overall, this study is meant to highlight how the synergism between the chemical structure and protonation process can be used to tailor the optoelectronic properties of imine-based materials and may be important for a further design of novel azomethines with desired properties.

## 2 Methods

### 2.1 Materials

Chloroform (98.5% vol) and acetone (99.5% vol) were purchased from ChemPur, *n*-hexane (99% vol) was purchased from Avantor Performance Materials, and *N*-methylpyrrolidone (99.5% vol) was purchased from Alfa Aesar, while acetonitrile (ACN) (anhydrous, 99.8% vol) and TFA were purchased from Sigma-Aldrich. All solvents were used as received, without any preceding purification.

**Figure 1:**
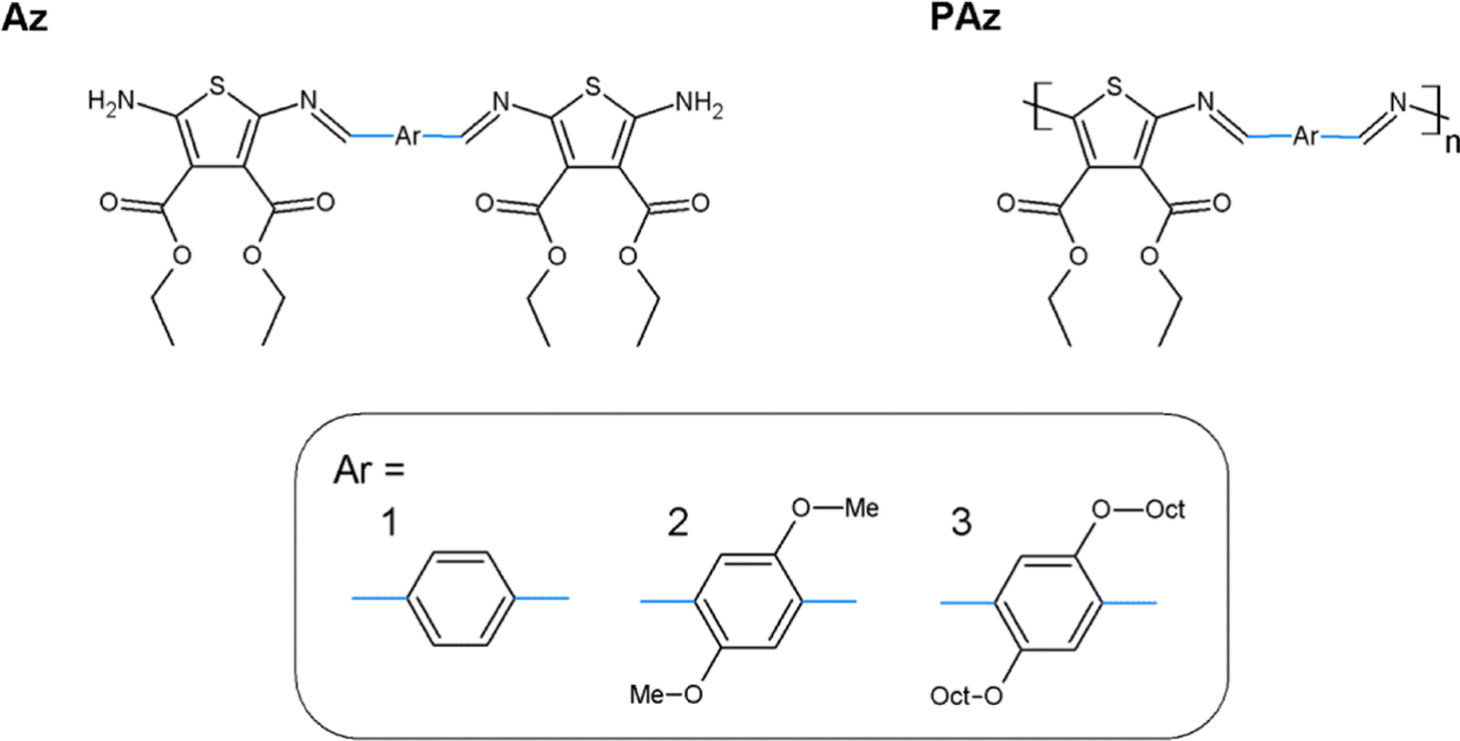
Chemical structures of investigated thiophene–phenylene-based Schiff bases with alkoxy side groups.

The chemical structures of the investigated model compounds (**Az**) and **PAzs** are shown in Figure 1. The model compounds are aromatic heterocyclic diamines consisting of a diimine system resulting from the condensation of an excess of diamine toward dialdehyde. The **PAz** compounds are products of polycondensation of equimolar amounts of diamine with dialdehyde. The variation in the chemical structure is similar in both types of compounds. The first one (1) consists of an unsubstituted benzene ring, the (**2**) phenylene group is substituted by methoxy side groups, while the (**3**) methyl groups in alkoxy side chains were elongated to *n*-octyl. All these compounds were characterized in detail in previous papers.^9,19^

The protonation process was carried out by adding appropriate amounts of the dopant into the solution of the neat imine (2:1, 4:1, and 10:1 molar ratios). To monitor the process in detail, TFA was added stepwise, increasing the molar ratio, followed by spectral measurements after each addition.

### 2.2 Characterization Techniques

#### 2.2.1.^1^H-NMR Spectroscopy

The ^1^H NMR spectra were recorded using the Avance II Ultrashield Plus spectrometer, which is operated at 600 MHz, using deuterated chloroform as a solvent and tetramethylsilane as an internal reference. The spectra were registered for each compound before and after addition of TFA.

#### 2.2.2 Optical Absorption and PL Spectroscopy

UV–vis absorption spectra were measured using a two-beam JASCO V-570 spectrophotometer. The absorption spectra of all compounds were registered in chloroform and two binary-solvent mixtures, namely, chloroform/*n*-hexane (CH/Hex) and chloroform/acetone (CH/A), to ensure a variation of the environmental dielectric constant. The concentration of solutions was maintained at 5 × 10^–5^ M for all investigated samples. The spectral range started at the cutoff wavelength of the solvents and ended at 800 nm. After the registration of spectra for neat compound solutions, TFA was added in three steps—through the introduction of 2 equiv (2 equiv), further 2 equiv (total 4 equiv), and finally 6 equiv (total 10 equiv) of the dopant, obtaining 2:1, 4:1, and 10:1 dopant/compound molar ratios, respectively. The absorption spectra were recorded after each of these steps. The concentration of all solutions during absorption measurements was considered constant due to the low amount of TFA added to the sample.

PL spectra were registered on a PerkinElmer LS 55 spectrometer. All compounds were investigated in the form of solutions, in the same solvent systems used for absorption studies, at a concentration of 5 × 10^–5^ M. The protonation process was monitored by PL spectroscopy upon the stepwise addition of TFA, measuring the spectra after each step. The excitation was performed at wavelengths corresponding to absorption maxima, designated from the electronic absorption spectra.

#### 2.2.3 Electrochemical Study

CV experiments were performed using a potentiostat/galvanostat (PG581, Uniscan Instruments). Experiments were carried in a standard one-compartment cell, in ACN, with 0.1 M tetrabutylammonium perchlorate as the supporting electrolyte, Pt wires as working and counter electrodes, and Ag/Ag^+^ as the reference electrode. Ferrocene was used as an external reference for calibration (*E*_onset_ = 0.35 V vs Ag/Ag^+^), and all potentials were referenced against it. The potentials were provided at room temperature, at the scan rate of 50 mV/s. The highest occupied molecular orbital (HOMO) values were calculated from the onset potentials, considering the absolute energy level of Fc/Fc^+^ as −5.10 eV versus vacuum.^43^ CV was also used to monitor the protonation of the molecules upon stepwise addition of the TFA dopant to the analyte solution. The cyclic voltammograms were recorded after each pot addition, and the changes in the redox potentials were followed.

## 3 Results and Discussion

### 3.1 Structural Studies

**Figure 2:**
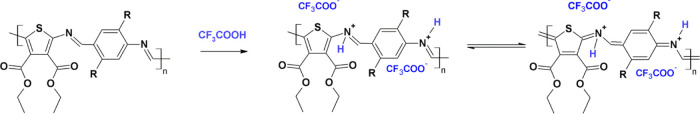
Proposed mechanism of PAz compound doping along with the envisaged resonance forms.

It is generally agreed that the mechanism of azomethine protonation includes bonding of the lone electron pair located at the imine nitrogen atom by a proton, with the formation of a positive charge that is stabilized by the counteranion.^44^ Accordingly, the protonation process of the investigated **PAz** compounds with TFA can be regarded as shown in Figure 2.

The protonation process was first investigated by ^1^H-NMR spectroscopy, to ensure that the compounds are stable under the acid doping conditions and check if any additional reactions may take place. The measurements were conducted only on **Az** model compounds, where it was much easier to follow the protonation process on the well-defined azomethine aromatic signals. The spectra of the pristine, unsubstituted azomethine model compound (**Az1**) (Figure S1) have revealed a singlet peak localized at 7.93 ppm, corresponding to imine proton, and another singlet due to aromatic protons at 7.76 ppm. Apart from them, signals from a negligible amount of the azomethine dimer and its aldehyde end group (10.05 ppm) were registered. The addition of TFA has resulted in the appearance of a new aldehyde proton signal, along with several new signals in the aromatic region between 8.24 and 7.92 ppm. Such a response for TFA addition clearly indicates the acid hydrolysis of the imine derivative toward the dimer (detachment of one thiophene diamine) and subsequently the constitutive monomers.

The spectra of alkoxy-substituted model compounds (Figure S2), however, have not indicated the occurrence of such degradation. The imine proton singlet, localized originally at 8.41 ppm, has shifted toward 8.52 ppm upon the addition of TFA. Similar behavior was observed for the aromatic proton signal, which has also revealed a shift from 7.54 to 7.66 ppm. Spectra of alkoxy-substituted model compounds (**Az2** and **Az3**) have not shown the rise of any new signals, unlike that observed for the unsubstituted azomethine (**Az1**). This suggests that such materials do not undergo any side reactions upon acid addition and are hydrolytically stable. The used amount of TFA was much higher than 10:1 molar ratio, as reflected by the distinct acid singlets at ∼10.50 ppm, proving the stability of alkoxy-substituted compounds.

### 3.2 Optical Measurements

The UV–vis absorption and PL spectra of the investigated compounds were registered for their solutions, using solvents or binary solvents with different dielectric constants, to vary the environment polarity. Since the only solvent in which all investigated compounds have dissolved was chloroform, the increase in the medium polarity was achieved using binary solvents. Their dielectric constants (ε) were calculated using a sum of the individual solvent’s dielectric constants weighted by their molar fractions, according to an equation,^45^ and the obtained values are gathered in Table 1.
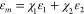
where ε_m_ is the dielectric constant of the binary solvent, ε_1_ and ε_2_ are the dielectric constant values for each solvent, and χ_1_ and χ_2_ are the solvent molar fractions.

**Table 1:** Dielectric Constant Values of Binary Solvents

solvent/binary solvent	ε_*m*_
chloroform/n-hexane (CH/Hex)	3.35
chloroform (CH)	4.81
chloroform/acetone (CH/Ac)	12.91

#### 3.2.1 UV–Vis Absorption

**Figure 3:**
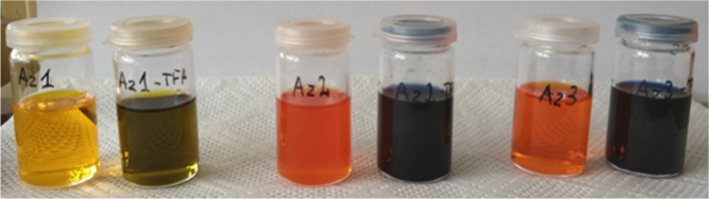
Pictures of **Az1**, **Az2**, and **Az3** solutions in chloroform before (left) and after (right) addition of 10 equiv of TFA.

The UV–vis absorption spectra of all investigated compounds’ solutions were recorded at room temperature, using chloroform and binary solvents to obtain a variable dielectric constant of the environment (Table 1). After registering spectra of the pristine compound solution, the protonation process was monitored, by adding subsequently 2 equiv (2:1 molar ratio), further 2 equiv (4:1 mol. ratio in total), and finally 6 equiv (10:1 mol. ratio in total) of TFA and recording spectra after each addition of the dopant. Introduction of TFA into the solution has resulted in distinct color changes of the studied solutions from yellow to green for **Az1** and from orange-red to dark-blue for **Az2** and **Az3** (Figure 3).

**Figure 4:**
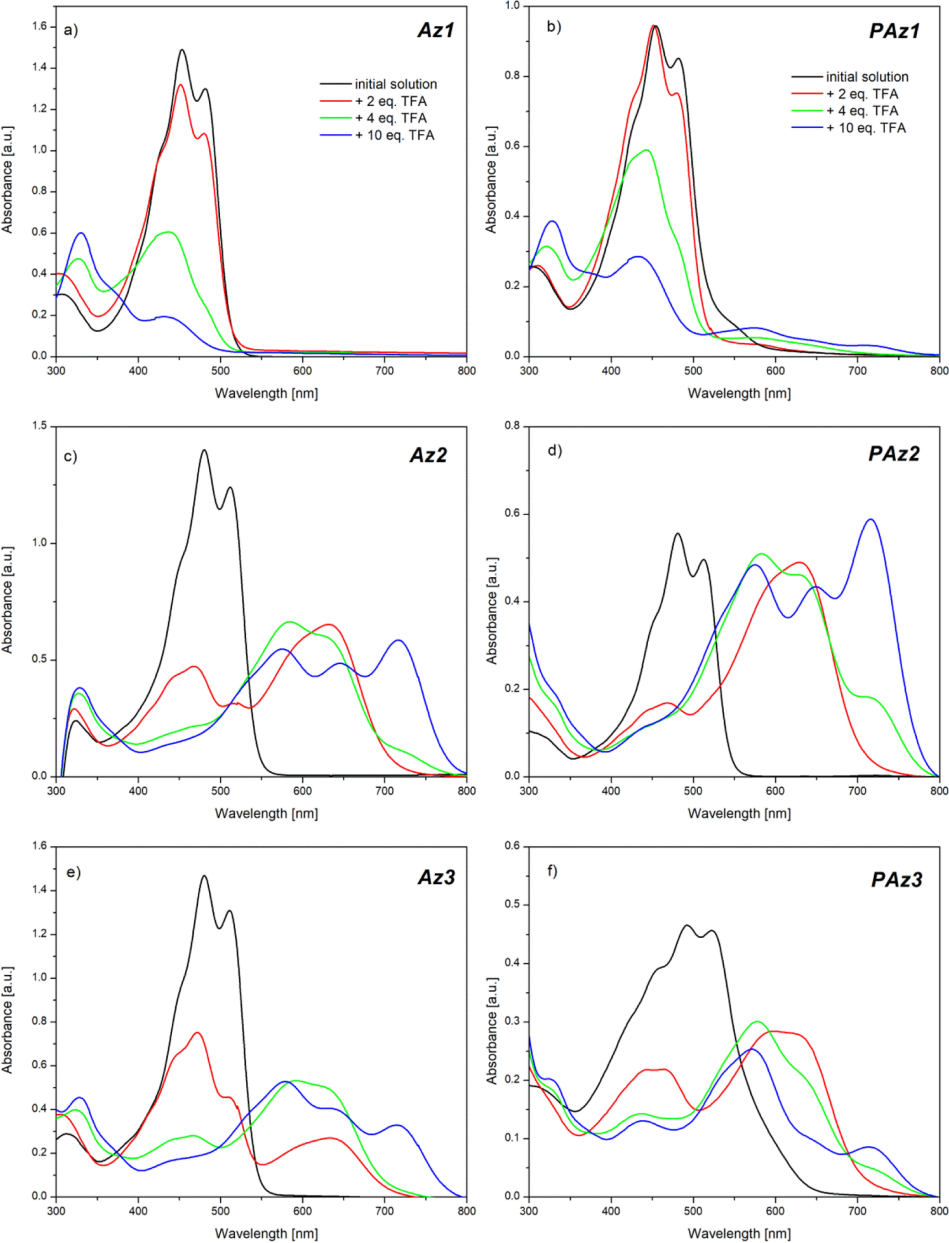
Absorption spectra of investigated model compounds (a,c,e) and polymers (b,d,f) in chloroform before (black lines) and after subsequent addition of TFA equivalents (color lines, according the legend seen in panels a,b).

The spectra for both pristine and doped model compounds and polymers were registered in the spectral range, starting at the cutoff wavelength of the solvent and ending at 800 nm (Figure 4).

The spectra of all pristine compounds in solution have revealed an absorption band localized between 450 and 630 nm, depending on the chemical structure and compound nature (**Az** or **PAz**), that can be associated with π → π* electron transitions.^9^ Each of these absorption bands has shown a vibronic structure, with the peaks being assigned according to the Franck–Condon principle.^46^ The presence of the dopant strongly influenced the absorption spectra of all compounds. The addition of the first amount of TFA (2 equiv) into the solution of the unsubstituted model compound (**Az1**) has resulted in a blue shift of the absorption band, together with a slight decrease in its intensity (Figure 4a). Subsequent introduction of the second dose of the dopant (4 equiv in total) has further decreased the intensity of the absorption band and shifted its position from 453 to 437 nm, simultaneously shifting the higher energy absorption band, connected with π → σ* or σ → π* electron transitions,^9^ from 303.5 to 327 nm. Finally, after addition of the last amount of the dopant (10 equiv in total), the observed absorption band has almost totally disappeared, indicating the degradation of the compound. Such behavior is consistent with the results obtained during ^1^H-NMR studies, which have proved that unsubstituted azomethine undergoes acid hydrolysis toward monomers. The protonation of the corresponding PAz (**PAz1**) has resulted in similar spectral changes in solution (Figure 4b). However, the decrease in the observed absorption band has proceeded slower, and after introduction of the second volume of the dopant, a weak broad absorption band, localized at 573 nm, has arisen. It may indicate that the degradation of **PAz1** is not complete at this content of TFA, while the remaining molecules would undergo protonation.

Substitution of the benzene ring with alkoxy side groups has enabled a stable protonation process to occur. The doping of the methoxy-substituted model compound (**Az2**) resulted in a decrease in the π → π* absorption band intensity, along with the formation of a new band localized at 631.5 nm after addition of 2 equiv of TFA (Figure 4c). Since ^1^H-NMR studies have shown that this imine does not undergo any side reaction, the new absorption band most probably originates from the partially protonated compound, while the remaining pristine imine still absorbs at 480 nm, however, with lower intensity. The increase in the dopant/compound molar ratio to 4:1 has resulted in the complete disappearance of the original absorption band, suggesting complete protonation of the molecule. The absorption band structure of the protonated compound has simultaneously developed through formation of an additional band, localized at 584.5 nm, and an inflection around 716 nm, which after addition of the final amount of the dopant (10 equiv) has evolved into a clear absorption band. Almost identical behavior during doping with TFA was observed when absorption studies were conducted with the corresponding oligoimine (**PAz2**) solution (Figure 4d) and also for octyloxy-substituted azomethine (**Az3**). Apparently, the length of the alkoxy side groups has not affected the position of the formed absorption bands. However, registered spectra of **Az3** have shown slower development of the absorption band, associated with the protonated moiety, and slower disappearance of the remaining pristine imine optical signal (Figure 4e). This could be explained by a reduced access of the acid molecules to the nitrogen atom in the imine bond, triggered by the steric effect of bulky octyloxy side groups, which may slightly hinder the protonation process. This is more obvious in the case of the corresponding polymer consisting of such substituents (**PAz3**), which displays in the registered spectra a not well-resolved vibronic structure of the absorption band assigned to protonated molecules and an important contribution from the absorption band of the neat polymer (Figure 4f).

**Figure 5:**
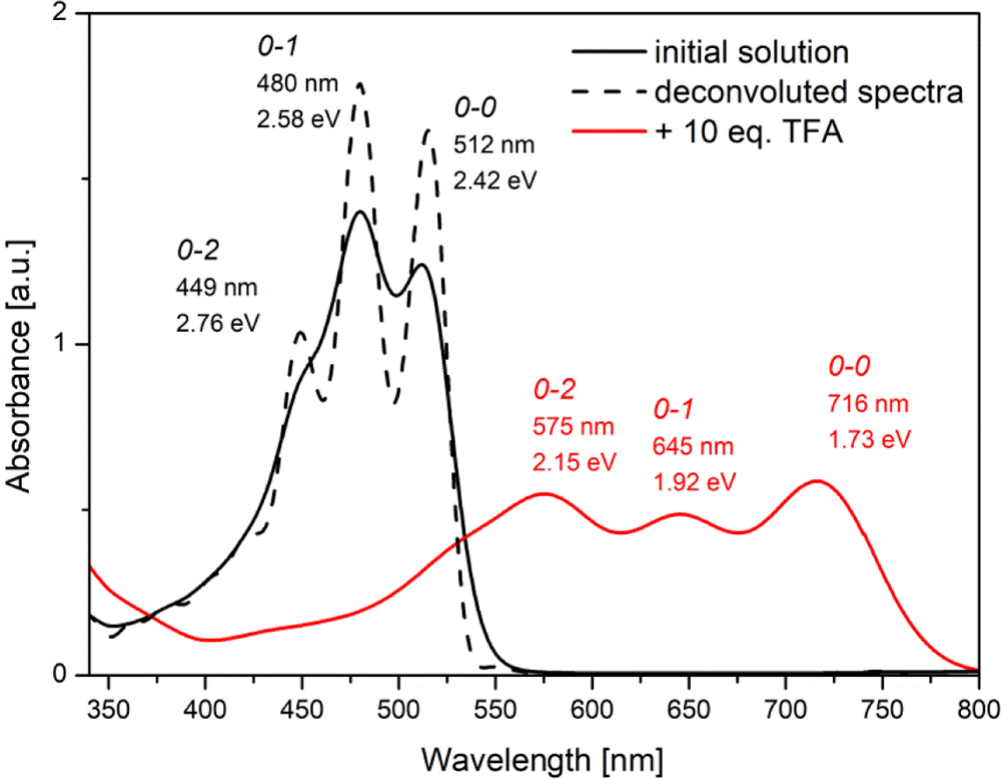
Absorption spectra of Az2 solution in chloroform before (black) and after (red) doping with TFA, together with assignment of vibronic peaks and their positions.

Since some of the π → π* absorption band vibronic peaks have not been very distinct, to find precisely the position of all bands, the second-derivative method was applied (i.e., the minimum of the second derivative of the absorption band corresponds to the absorption maximum). To ensure the consistency of the data, all remaining band positions were designated in a similar manner, and then, the vibronic progression of bands has been deconvoluted with the modified Fourier self-deconvolution and finite response operator methods.^47^ These vibronic bands have revealed the electron–phonon interaction connected with the aromatic ring stretching mode. For example, the spectrum of **Az1** after deconvolution is shown as a dotted line in Figure 5. The energy differences between neighboring peaks were found in the range of 0.16–0.22 eV for all studied compounds, with the most intense peaks corresponding to the lowest-energy transitions between 0–0 and 0–1 vibronic levels.^19^ The analysis of protonated molecule bands’ positions in terms of energy has highlighted a difference between them of ∼0.2 eV (Figure 5), which clearly indicates that these are vibronic peaks of the absorption band, connected with π → π* electron transitions in the protonated imine.

**Figure 6:**
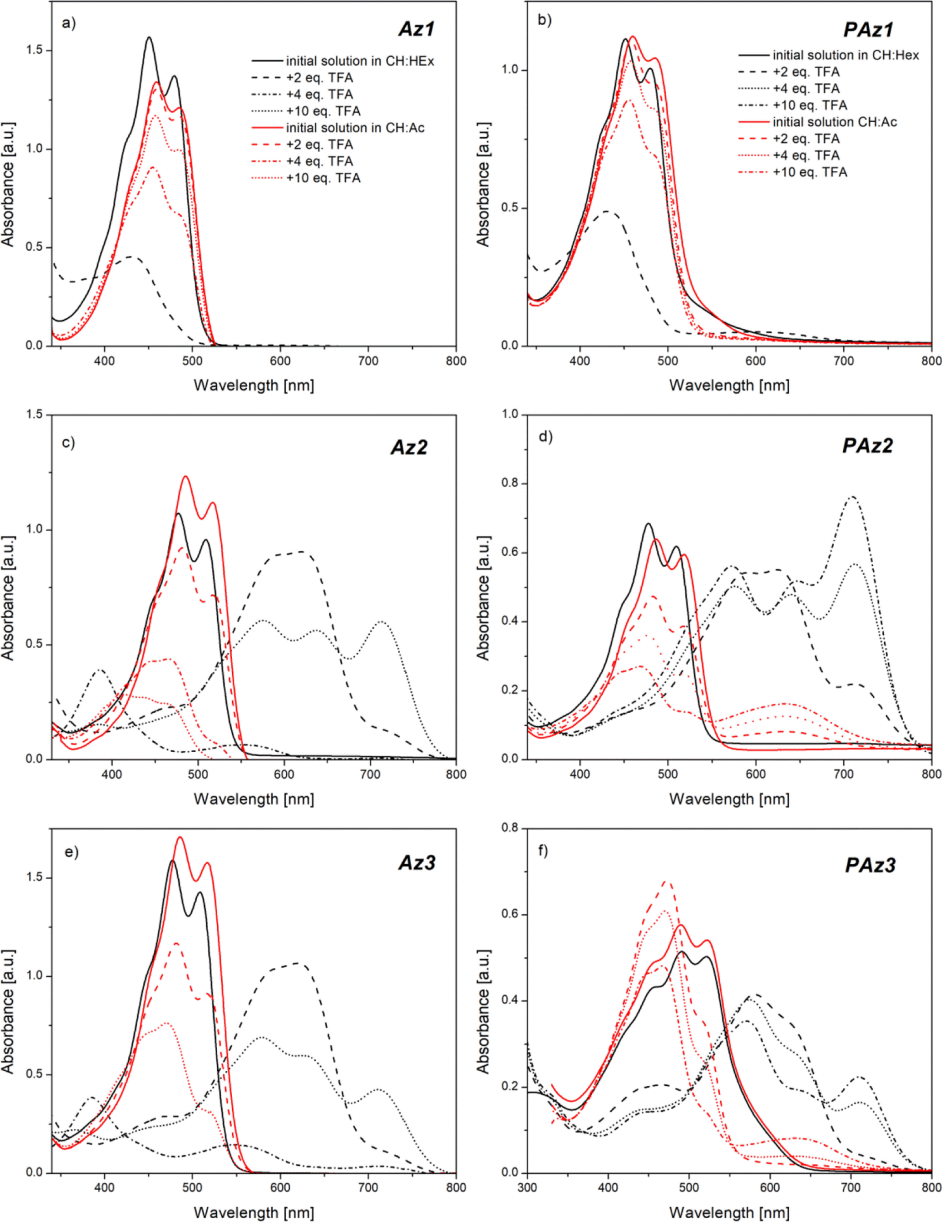
Absorption spectra of investigated model compounds (left) and corresponding polymers (right) in binary solvents of chloroform/*n*-hexane (CH/Hex)—(black) and chloroform/acetone (CH/Ac)—(red) before and after the subsequent doping with 2, 4, and 10 equiv of TFA (various line styles).

Apart from the influence of the chemical structure of compounds on the protonation process, also, the impact of solvent polarity on the optical response induced by the acid doping was investigated (Figure 6).

The dielectric constant of utilized binary solvents (Table 1) has significantly impacted the protonation process and its optical signature. The UV–vis spectra recorded in chloroform/*n*-hexane (solid lines), with the lowest ε, indicated a similar behavior to that registered in chloroform for all compounds. Also, the hydrolysis of unsubstituted compounds **Az1** and **PAz1** (Figure 6a,b) has proceeded almost instantly, at 2:1 molar ratio of the dopant/compound in this binary solvent. Even alkoxy-substituted model compounds **Az2** and **Az3** (Figure 6c,e) have undergone degradation in this medium after the introduction of a too high amount of dopant (10 equiv in total). However, the protonation of **PAz2** and **PAz3** in these binary solvents has proceeded faster, when the vibronic structure of the absorption band evolved into a more pronounced one than in chloroform (Figure 6d,f). Utilization of a more polar binary solvent (dashed lines), for example, chloroform/acetone (CH/Ac) having a higher dielectric constant than chloroform, significantly hindered the doping process. Although the spectra of unsubstituted compounds **Az1** and **PAz1** (Figure 6a,b) have revealed a subsequent decrease in the absorption band intensity; correlated with acidic hydrolysis, the observed hypochromic effect proceeded much slower than in less-polar solvents. It is worth noticing that the electronic spectra of remaining, alkoxy-substituted compounds have not shown any new band formation, but rather a subsequent decrease in the intensity of the absorption band of neat imines is observed. All these results suggest that the protonation process is supported only in non-polar solvents with low dielectric constants. The reason why a more polar environment has probably hampered the acid doping process is believed to be connected with the less-stabilized protonated imine molecules in polar media. However, in the case of polymers, a weak absorption band centered at approx. 640 nm appeared, which may suggest that PAz protonation occurred to a low extent even in a polar environment.

#### 3.2.2 Photoluminescence

Investigation of the emissive properties of the studied compounds in the neat and protonated forms was conducted in the same solvent/binary solvents used for the absorption measurements. The PL spectra were first registered in chloroform after excitation with wavelengths corresponding to the vibronic peaks of the π → π* absorption band observed after addition of the corresponding amount of the acid dopant. In the case of azomethine model compounds, the PL response was analyzed only after an instant addition of 10 equiv of TFA, while in the case of polyimines, the PL emission was registered after sequential doping with TFA.

Addition of the dopant molecules into the unsubstituted compound (**Az1** and **PAz1**) chloroform solutions has caused the PL increase along with a hypsochromic shift (up to 29 nm) of the PL maxima (Figure S3). Usually, molecules consisting of azomethine moieties are known for a suppressed PL due to the internal conversion processes involving bond rotation^48^ that may be reactivated through protonation when the rotational barrier increases.^39^ However, since previous ^1^H-NMR and absorption studies have proved the occurrence of acid hydrolysis of these compounds, this enhanced emission is considered to originate from the formed dimers or monomers rather than from the protonated molecules.

**Figure 7:**
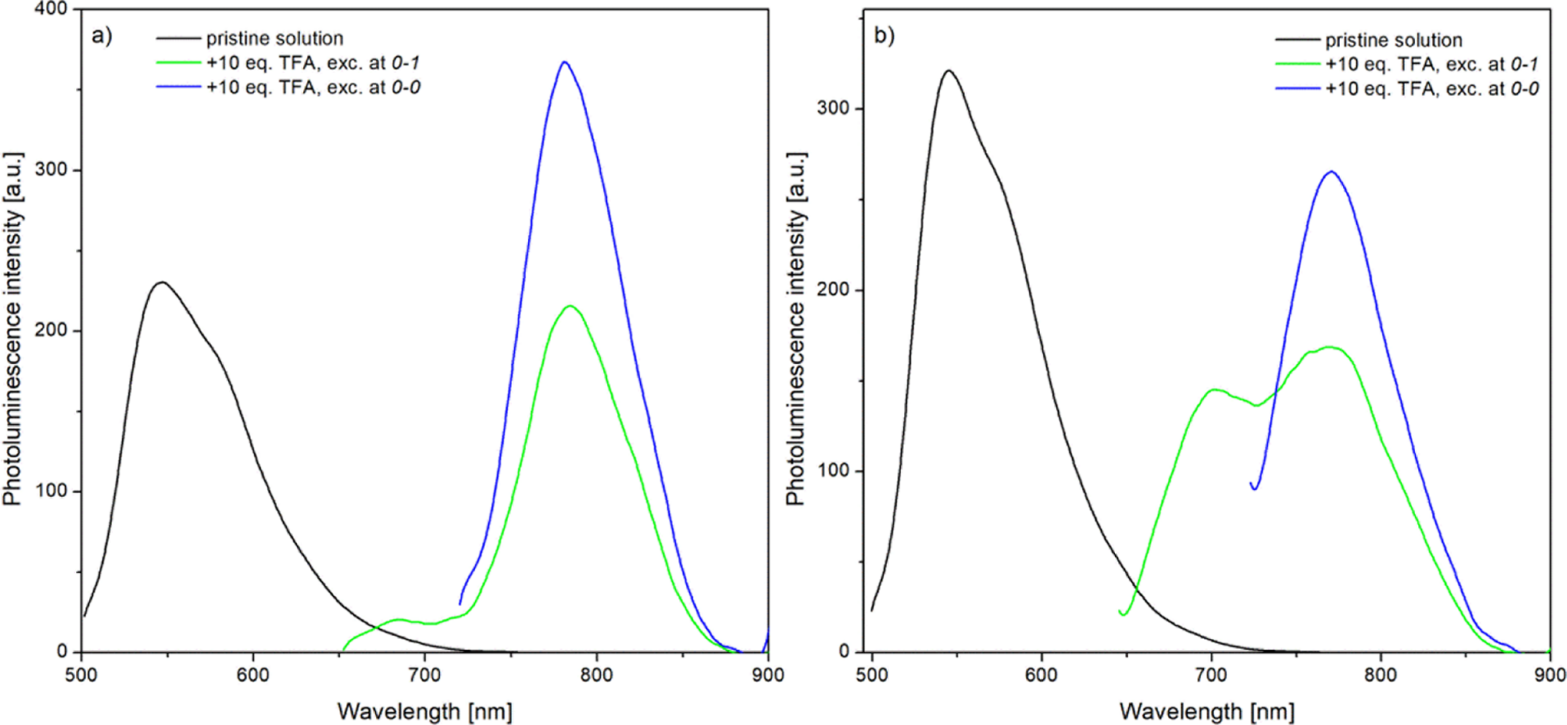
PL spectra of chloroform solutions of azomethines: Az2 (a) and Az3 (b) before and after protonation with 10 equiv of TFA, upon excitation with various wavelengths.

On the other hand, the incorporation of alkoxy side groups in the chemical structure of the remaining compounds has allowed protonation to occur, and hence, any variation in PL is expected to be induced by the formation of a positive charge at the azomethine nitrogen center. Doping of methoxy (**Az2**)- and octyloxy (**Az3**)-substituted azomethines has resulted in a bathochromic shift of the emission band (due to excitation with 0–0 vibronic band wavelength) from 558 nm for pristine compounds up to 781 and 771 nm for the protonated forms of **Az2** and **Az3**, respectively (Figure 7), being accompanied by a slight intensity variation.

Since the length of the alkyl units does not influence the electronic structure of the compounds,^9,19^ the registered difference between the emission band positions of these two related compounds may be due to a non-complete protonation process of **Az3**, caused by the steric hindrance induced by the bulky octyl groups. Such a hypothesis would be in agreement with the results of absorption measurements, where the absorption bands of octyloxy-substituted molecules developed slower than those of their methoxy counterparts. A similar conclusion has been made during PL studies performed at various excitation wavelengths, corresponding to the vibronic peak positions (Figure 7). The PL of **Az2** registered upon excitation at both 0–1 and 0–0 vibronic peak wavelengths consisted of one emission band, localized at 781 nm, originating from fully protonated molecules (Figure 7a). The only difference is related to their intensity, which was considerably enhanced at the 0–0 vibronic peak wavelength excitation. Due to a partial doping process of the octyloxy-substituted compound (**Az3**), excitation at the wavelength of 0–1 vibronic peak has resulted in a spectral profile with two emission (Figure 7b) peaks, at 704 and 771 nm, which correspond to partially and fully protonated molecules, respectively. Only excitation with lower energy, corresponding to the 0–0 vibronic peak, resulted in more intense emission at 771 nm, however, of the lower intensity, due to a smaller contribution of the fully protonated molecules.

**Figure 8:**
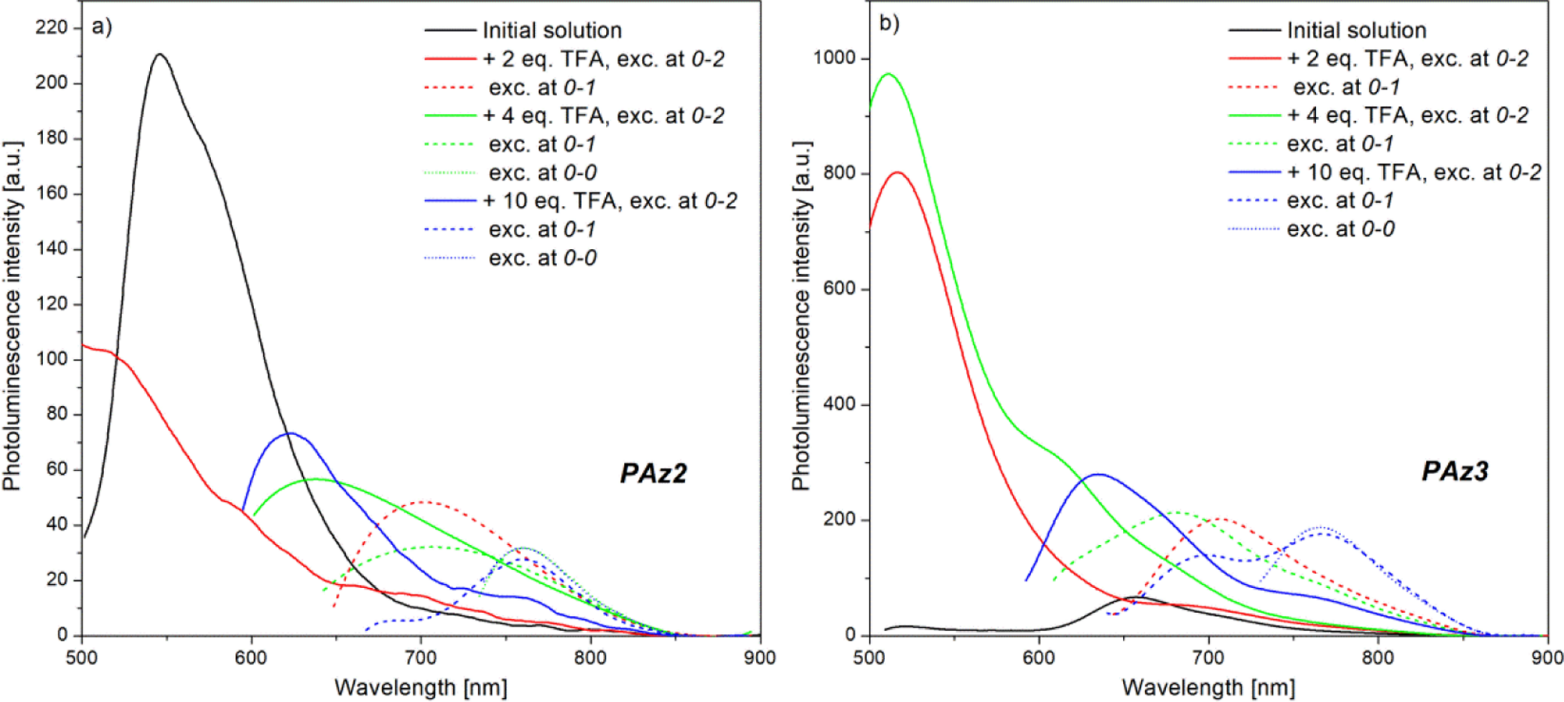
PL spectra of PAz2 (a) and PAz3 (b) solutions in chloroform, upon excitation with various wavelengths during sequential addition of the dopant.

The development of PL bands upon protonation was even more visible during sequential doping of polyimines with TFA (Figure 8).

Sequential addition of the dopant into investigated solutions of **PAz2** and **PAz3** has resulted in a stepwise shift of the emission band, which is actually dependent on the excitation wavelength. Excitation with energy corresponding to the 0–2 vibronic peak of **PAz2** doped with 2 equiv of TFA (Figure 8a) has resulted in a PL emission decay of the pristine polymer in chloroform, which turned on after subsequent introduction of the next amount of the dopant (4 equiv in total), however, red-shifted. As the proton-induced change is saturated after addition of the last TFA volume (10 equiv in total), the PL spectrum became well resolved, with a maximum at 623 nm. This shift of fluorescence spectra in response to protonation as a consequence of the pH increase was considered to be triggered by the degree of interaction and extent of charge transfer complex formation between the acid and the nitrogen atom of the imine bond in the polymer.^49^ When lower energies, corresponding to the 0–1 and 0–0 vibronic peaks, were used for photoexcitation, the PL bands of partially protonated **PAz2** molecules (at 2 and 4 equiv of dopant) have shifted toward longer wavelengths, so that at complete protonation (10 equiv in total), the emission has taken place in the low-energy spectral range, with a PL band centered at 760 nm (Figure 8a). However, the emission from the protonated molecules is very low, compared to that of native molecules, most likely due to the deactivation of the singlet excited state through photoinduced electron transfer.^50^ Nevertheless, protonated **PAz2** molecules may develop H bonds at the N atom of the azomethine center that could favor charge separation and fluorescence quenching.^51^

A completely different PL behavior has been registered in the case of **PAz3** at partial protonation with TFA. Excitation with wavelengths characteristic for non-protonated molecules, which are still present in the **PAz3** solution at addition of 2 and 4 equiv of TFA, has resulted in a very intense emission band localized at 517 nm, blue-shifted in respect to the PL band of the pristine **PAz3** in chloroform solution (Figure 8b). Since no acid hydrolysis has taken place as demonstrated by ^1^H-NMR studies, this PL response might originate from the intrinsic fluorescence, which was released after partial protonation. Such behavior agrees well with previous reports on PAz protonation that demonstrated protonation-induced fluorescence enhancement as a consequence of the rotational barrier increase around the imine bond.^39^ Nevertheless, these findings also support the previous statement, according to which the protonation of octyloxy-substituted molecules has developed slower than in the case of their methoxy counterparts that were completely protonated at 2:1 molar ratio of dopant/compound. However, in the presence of 10 equiv of dopant, only the PL band, characteristic for the fully protonated molecules due to the photoinduced electron transfer between TFA and the imine unit, has been recorded at a considerably reduced intensity as compared to that of the intrinsic fluorescence band. As for the excitation with lower energies, corresponding to 0–1 and 0–0 vibronic bands of the protonated molecules, the PL behavior was similar to that encountered for **PAz2**. The charge transfer emission band has undergone a red shift, and after addition of the last amount of the dopant, it was split into two absorption bands with maxima at 763 and 705 nm (Figure 8b). However, only the excitation at the wavelength characteristic for the 0–0 absorption peak has resulted in the PL emission at 766 nm, considered to be released by the fully protonated molecules, while the higher energy-induced PL emission is considered to be generated by an incomplete protonation process due to the presence of the steric hindrance that has endowed the analyte sample with both partially and completely protonated **PAz3** molecules.

Emission spectra of alkoxy-substituted compounds, taken in binary solvents of various dielectric constants, have revealed a positive solvatofluorochromism, suggesting a more effective solvation of the excited molecule and subsequently a better stabilization of the excited states in more polar solvents, similar as that in ref (19). Apart from that, solvent polarity has affected the protonation process in a similar manner as that during absorption studies. PAz PAz1 has hydrolyzed in a mixture of chloroform/*n*-hexane, which is visible as a hypsochromic shift of the PL band (Figure S4a), while the more polar binary solvent CH/Ac has hindered the hydrolysis and allowed the PL intensity to increase (Figure S4b). In a mixture of chloroform and *n*-hexane, the doping of alkoxy-substituted imines has proceeded much faster, and the emission bands were more intense, with a similar profile as that found in chloroform. Instead, in a polar binary solvent consisting of chloroform and acetone, the protonation occurred to some extent as reflected by the decrease in the native PL emission in the case of **PAz2** and a significant increase in the case of **PAz3** (Figure S5). The opposite effects registered for these two related molecules can be rationalized in terms of the singlet excited-state deactivation through photoinduced electron transfer and the rotational barrier increase around the imine bond.

### 3.3 Cyclic Voltammetry

The electrochemical measurements of the protonated imine compounds were accomplished using CV, at a scan rate of 50 mV/s. All experiments were performed for imines as solute analytes in ACN, registering the cyclic voltammograms (CVs) for both neat imine solutions and after each subsequent addition of the TFA dopant.

**Figure 9:**
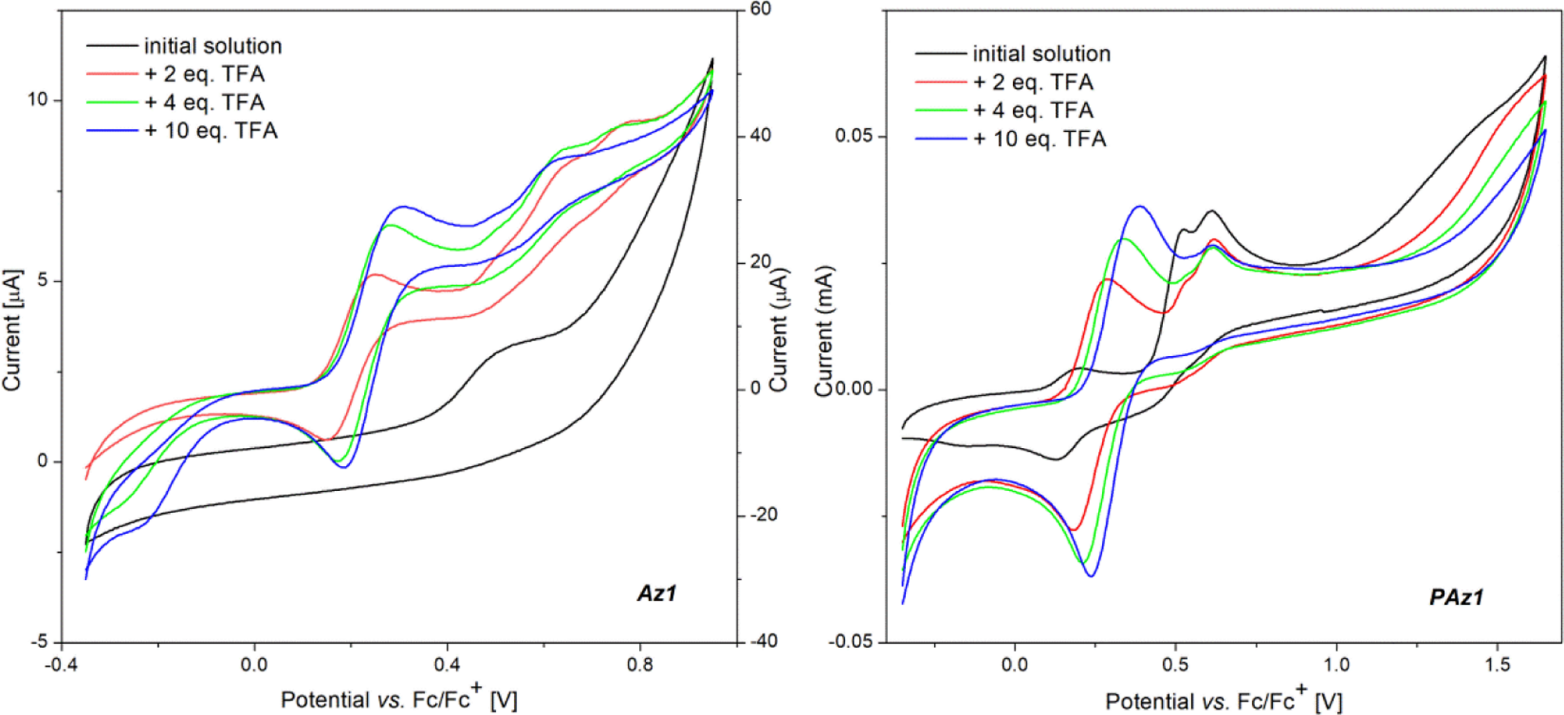
Voltammograms of **Az1** (a) and **PAz1** (b) solutions in ACN before and upon protonation.

Before assessing the electrochemical behavior of protonated imine molecules in ACN solution, the influence of the molecular structure on the oxidation potentials of the pristine compounds as individual molecules was surveyed. As previously reported for the **PAz1**-modified ITO electrode,^19^ two oxidation peaks associated with the oxidation of ester-substituted thiophene were recorded for **PAz1** in solution, with the onset of the oxidation process (*E*_onset_^ox^) at 0.39 V versus Fc/Fc^+^ (Figure 9).

Although the corresponding model compound **Az1** has a reduced conjugation degree compared to **PAz1**, its oxidation started at lower potentials, 0.37 V versus Fc/Fc^+^, owing to the electron donor character of the NH_2_ groups. When 2 equiv of TFA was added to the solution of **PAz1**, one of the peaks corresponding to the oxidation waves of ester-substituted thiophene slightly shifted from the original potential values but significantly decreased, with eventual disappearance, upon doping with the total TFA/compound ratio of 10:1. Meanwhile, a new band has evolved at lower potentials that can be assigned to a reversible oxidation process of the NH_2_ groups of the formed thiophene diamine monomer upon hydrolysis of the azomethine bond, in line with previous findings. As more NH_2_ groups appear in the system, when the doping advances, this peak intensity increases and slightly shifts to more positive potential values. This process is accompanied by the onset potential decrease from 0.39 and 0.37 V versus Fc/Fc^+^ to 0.11 V versus Fc/Fc^+^ (at 2:1 TFA/Az molar ratio), for **Az1** and **PAz1**, respectively.

**Figure 10:**
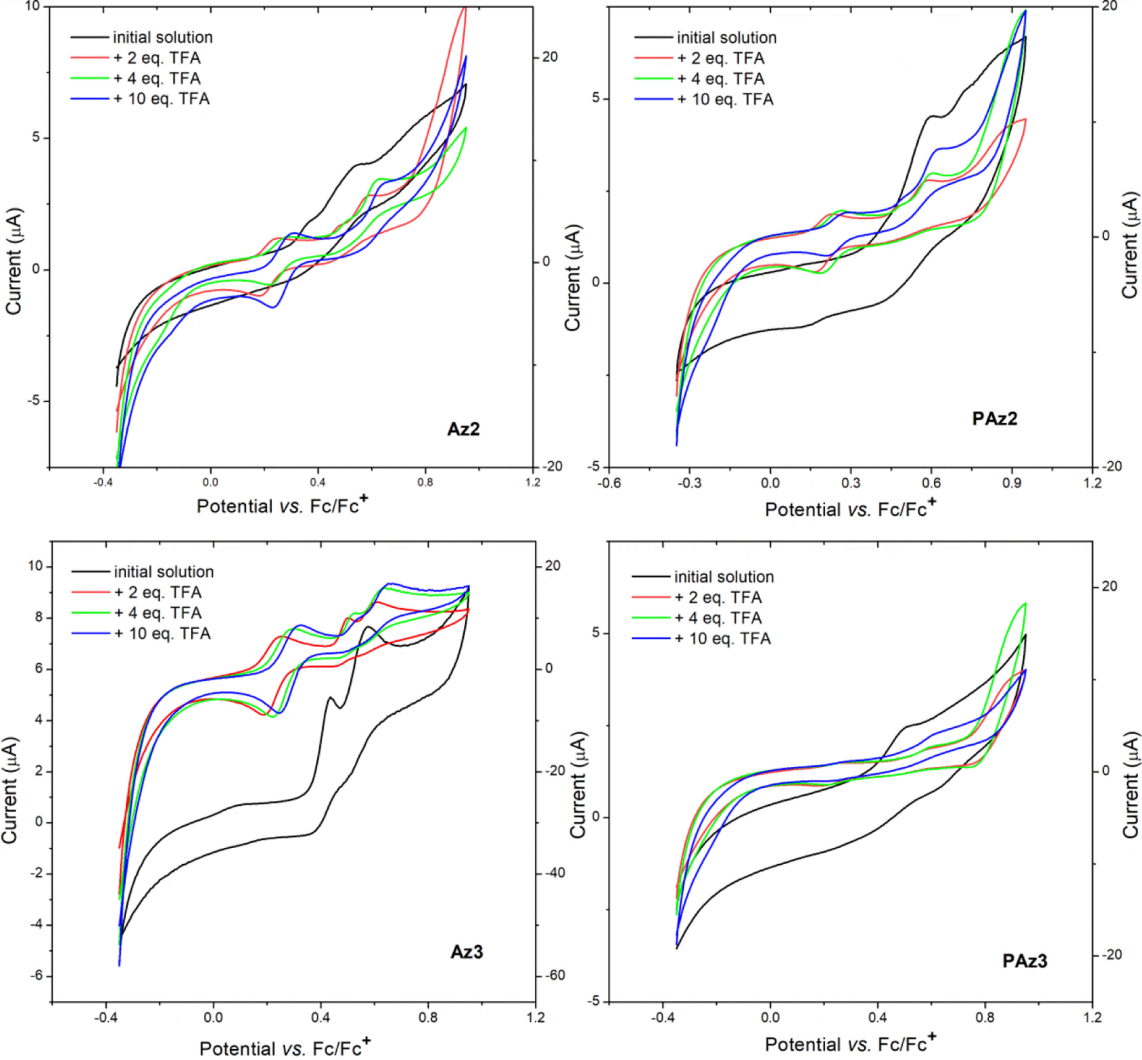
Voltammograms of **Az2** (a), **PAz2** (b), **Az3** (c), and **PAz3** (d) in ACN solutions before and upon protonation.

Substitution of the benzene rings with either methoxy or octyloxy groups in **PAz2** and **PAz3,** respectively, has slightly lowered the onset potential, as expected, since these groups increase the electron abundance on the thiophene ring, facilitating an easier oxidation. The oxidation of this series of Schiff bases is clearly not reversible. One of the reasons for this should be the electron-deficient nature of the thiophene substituents, namely, ester groups and imine bonds. Once the electron is released by the thiophene ring, it can be trapped by these groups, so that the formed radical cations cannot reach the neutrality when the current is reversed to 0 V. The presence of amino groups in Az compounds has also resulted in similar onset potential values of **Az2-PAz2** (0.31 vs 0.35 V) and **Az3-PAz3** (0.33 vs 0.36) pairs (Figure 10), despite various conjugation degrees.

As for the imines **PAz2** and **PAz3**, they have shown similar response to protonation. A new oxidation peak has appeared at 0.23 V versus Fc/Fc^+^ (**PAz2**) or 0.28 V versus Fc/Fc^+^ (**PAz3**) in the presence of 2 equiv of TFA, which is slightly shifted to higher values as protonation advanced (Figure 10b,d). Meanwhile, the existing peaks at 0.42/0.59 V versus Fc/Fc^+^ for **PAz2** and 0.50 V versus Fc/Fc^+^ for **PAz3** in the neutral form were found for the protonated molecules at 0.50/0.62 V versus Fc/Fc^+^ for **HPAz2**^**+**^ and 0.60 V versus Fc/Fc^+^ for **HPAz3**^**+**^, at 10:1 TFA/PAz molar ratio. This is expected, considering that upon protonation, the electron-withdrawing character of azomethine increases, and the thiophene and alkoxy units will oxidize with much difficulty due to the stronger depleting effect of the protonated azomethine moiety. The evolved oxidation peak that proved to be reversible can be assigned to the oxidation of the protonated molecules (**HPAz**^**+**^), probably prompted by the electron-donating effect of thiophene and alkoxy units. According to a similar mechanism proposed for the oxidation of protonated tetrathiafulvalene,^52^ it may be assumed that the oxidation of **HPAz**^**+**^ has led to unstable **HPAz**^**2+•**^ that has dissociated in **HPAz**^**+•**^and H^+^. Once the potential scan direction is reversed, these radical cations reach the neutrality, restoring the native molecules in a reversible process. Owing to this oxidation process, the **HPAz2**^**+**^ and **HPAz3**^**+**^ are much easier to oxidize compared to the unprotonated molecules, with the (*E*_onset_^ox^) shifted to 0.21 V versus Fc/Fc^+^ (Table 2).

**Table 2:** Electrochemical Data and Energy Levels of Oligo- and PAzs Upon Protonation^a^

	initial solution		+2 equiv of TFA		+4 equiv of TFA		+10 equiv of TFA	
name	*E*_onset_^ox^ [V]	*E*_HOMO_ [eV]	*E*_onset_^ox^ [V]	*E*_HOMO_ [eV]	*E*_onset_^ox^ [V]	*E*_HOMO_ [eV]	*E*_onset_^ox^ [V]	*E*_HOMO_ [eV]
Az2	0.31	–5.41	0.16	–5.26	0.18	–5.28	0.20	–5.30
Az3	0.33	–5.43	0.15	–5.25	0.19	–5.29	0.21	–5.31
PAz2	0.35	–5.45	0.14	–5.24	0.18	–5.28	0.21	–5.31
PAz3	0.36	–5.46	0.18	–5.28	0.19	–5.29	0.21	–5.31

a *E*_onset_^ox^—the onset oxidation potential; *E*_HOMO_ = −e^–^ (5.1 + *E*_onset_^ox^) according to ref (43).

In the case of the corresponding model compounds, **Az2** and **Az3**, the electrochemical behavior was similar (Figure 10a,c), while the reversibility of the oxidation process of the protonated forms is even more obvious. The variation in the *E*_onset_^ox^ followed the same trend as in the case of **PAz2** and **Paz3**, being registered at similar values (0.20 and 0.21 V vs Fc/Fc^+^ for **Az2** and **Az3**, respectively). Unfortunately, we could not discriminate between complete or incomplete protonation processes at 10:1 molar ratio of TFA toward **Az2** and **PAz2** or **Az3** and **PAz3**, since on the proposed mechanism, the oxidation process involves the regeneration of the native imine molecules.

An attempt to record the CV diagrams of the studied protonated molecules in the cathodic region has failed since no electrochemical activity was present under the chosen experimental conditions.

The obtained values of *E*_onset_^ox^ have allowed us to calculate the energies of the HOMOs according to the measured potential of ferrocene used for calibration versus the reference Ag/Ag^+^ electrode (*E*_onset_ = 0.35 V) (Table 2).^43^ Initial compounds due to high energy of the HOMO level (>−5.5 eV) have revealed a *p*-type semiconductor character.^53^ The HOMO values estimated after the protonation have suggested that doping with TFA improved the hole injection properties of these imines by lowering the HOMO level and overall led to a better *p*-type behavior.

## 4 Summary and Conclusions

This study presents the results of spectroscopic and electrochemical investigations on tuning the optoelectronic properties of some PAzs and their model compounds upon protonation with TFA. The effect of chemical structure variation, which is the substitution with alkoxy side groups and their length, on the doping process has been considered. Moreover, the influence of solvent polarity on the protonation has been studied.

The results of this work proved that non-substituted imines undergo hydrolysis instead of the protonation process, resulting in a decrease in the absorption band intensity together with their blue shift and a decrease in the potential required for the beginning of the oxidation process. ^1^H-NMR spectra of alkoxy-substituted compounds, however, have shown that these compounds do not undergo hydrolysis or any other reaction upon introduction of TFA. Their optical spectra have indicated a distinctive bathochromic shift (higher than 200 nm) of the absorption band connected with π → π* electron transitions. Although the spectra of model azomethines have shown that the length of alkoxy substituents has not affected the positions of absorption bands of protonated molecules, the evolution of these electronic spectra has proceeded slower for compounds consisting of octyloxy side groups. Such bulky alkyl groups have probably exerted a steric hindrance, hindering the access of acid molecules to the imine bonds and, as a result, the whole protonation process. The PL spectra have also shown bathochromic shifts of the emissive bands upon protonation but without any significant increase in their intensity at complete protonation. It has been noticed that at partial protonation, the low fluorescence of native imines was turned on. Electrochemical studies of these alkoxy-substituted imines have indicated a better *p*-type behavior after protonation, induced by the capability of the protonated form to oxidize in ACN and to generate the native molecules.

Apart from the influence of the chemical structure, this study has also considered the effect of polarity of the utilized solvent on the doping process. It has been shown that the protonation proceeded in high yields in solutions with low dielectric constants (chloroform and a mixture of chloroform and *n*-hexane), while more polar binary solvents consisting of chloroform/acetone have not allowed the doping process to occur to a high extent.

This paper offers a new insight into the influence of the chemical structure and dielectric constant of the environment on the doping process using TFA. Stepwise addition of the dopant allowed monitoring the individual phases of the protonation process. Such results might be useful during designing novel compounds for systems focused on halochromic applications.

## Supporting Information Available

NMR spectra of azomethine model compounds upon protonation; PL spectra of PAz1 in chloroform; and PL spectra in binary solvents (PDF)
