# Supplementary material for: Effect of Protonation on Optical and Electrochemical Properties of Thiophene–Phenylene-Based Schiff Bases with Alkoxy Side Groups
Source: J Phys Chem B. 2021 Jul 27;125(30):8588–600. doi: 10.1021/acs.jpcb.1c05390 (PMC8389983; doi:10.1021/acs.jpcb.1c05390)
Supplement: Supplementary file 1 — jp1c05390_si_001.pdf [file jp1c05390_si_001.pdf]

SUPPORTING INFORMATION

The Effect of Protonation on Optical and Electrochemical Properties of Thiophene-Phenylene Based Schiff-Bases with Alkoxy Side Groups

Paweł Nitschke[1], Bożena Jarząbek*[1], Andra-Elena Bejan[2] and Mariana-Dana Damaceanu[2]

[1]*Centre of Polymer and Carbon Materials, Polish Academy of Sciences, 34 M. Curie-Skłodowska Str., 41-819 Zabrze, Poland*;

[2]*Electroactive Polymers and Plasmochemistry Laboratory, "Petru Poni" Institute of Macromolecular Chemistry, Aleea Grigore Ghica Vodă nr. 41A, Iași 700487, Romania*

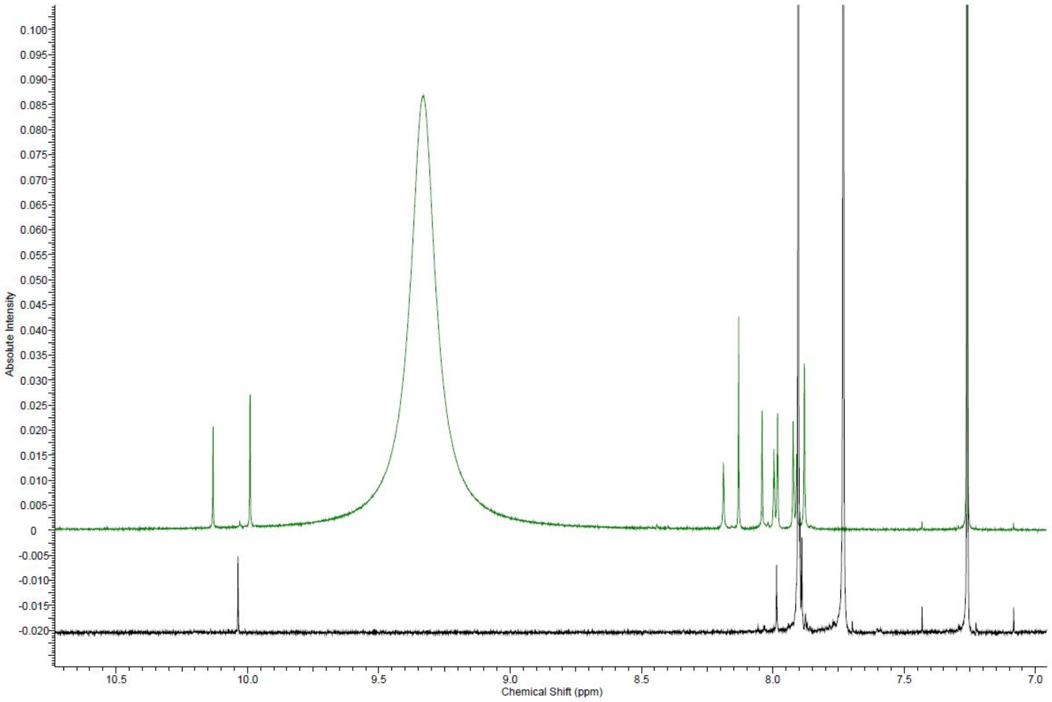

Figure S1.^{1}H-NMR spectra registered during protonation of **Az1** in $CDCl_3$. (black line –before protonation; green line – after addition of TFA).

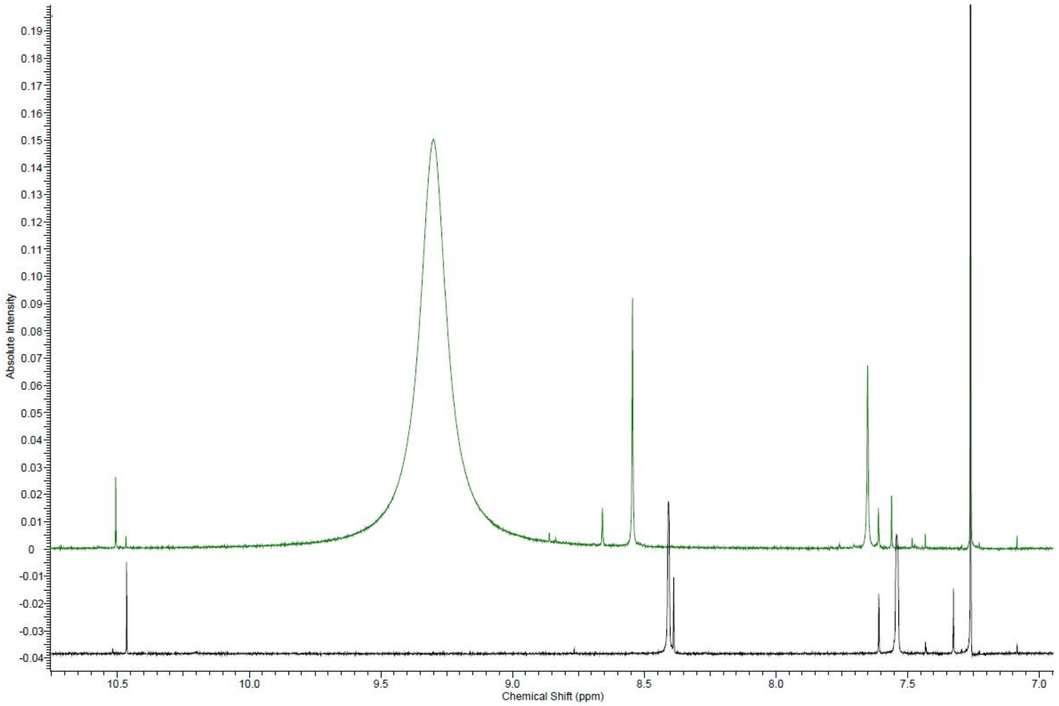

Figure S2.^{1}H-NMR spectra registered during protonation of **Az3** in $CDCl_3$. (black line –before protonation; green line – after addition of TFA).

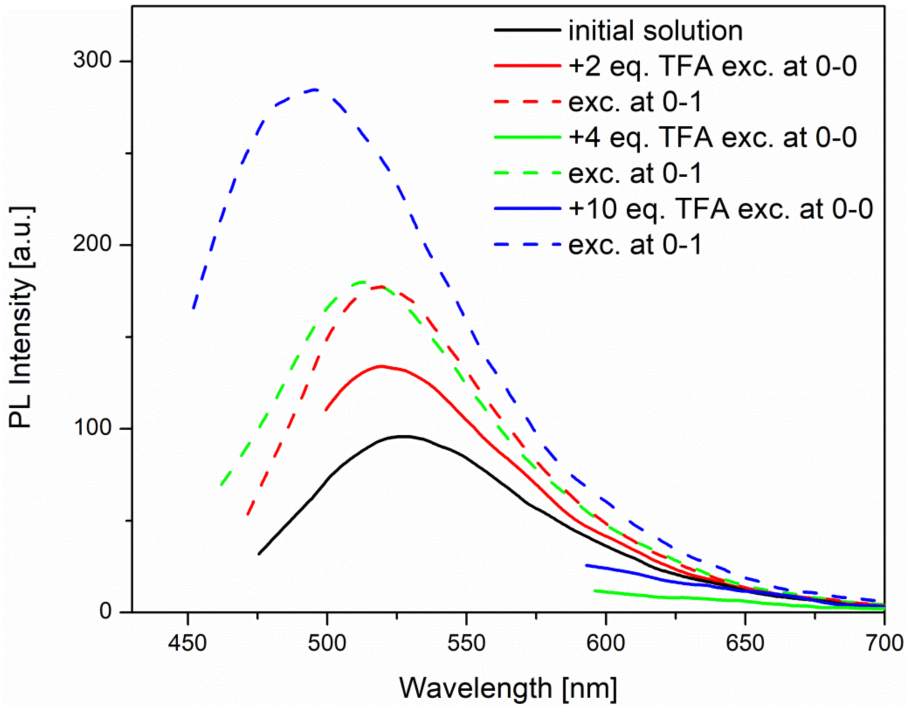

Figure S3. Photoluminescence spectra of PAz1 solution in chloroform, upon excitation with various wavelengths during sequential addition of dopant.

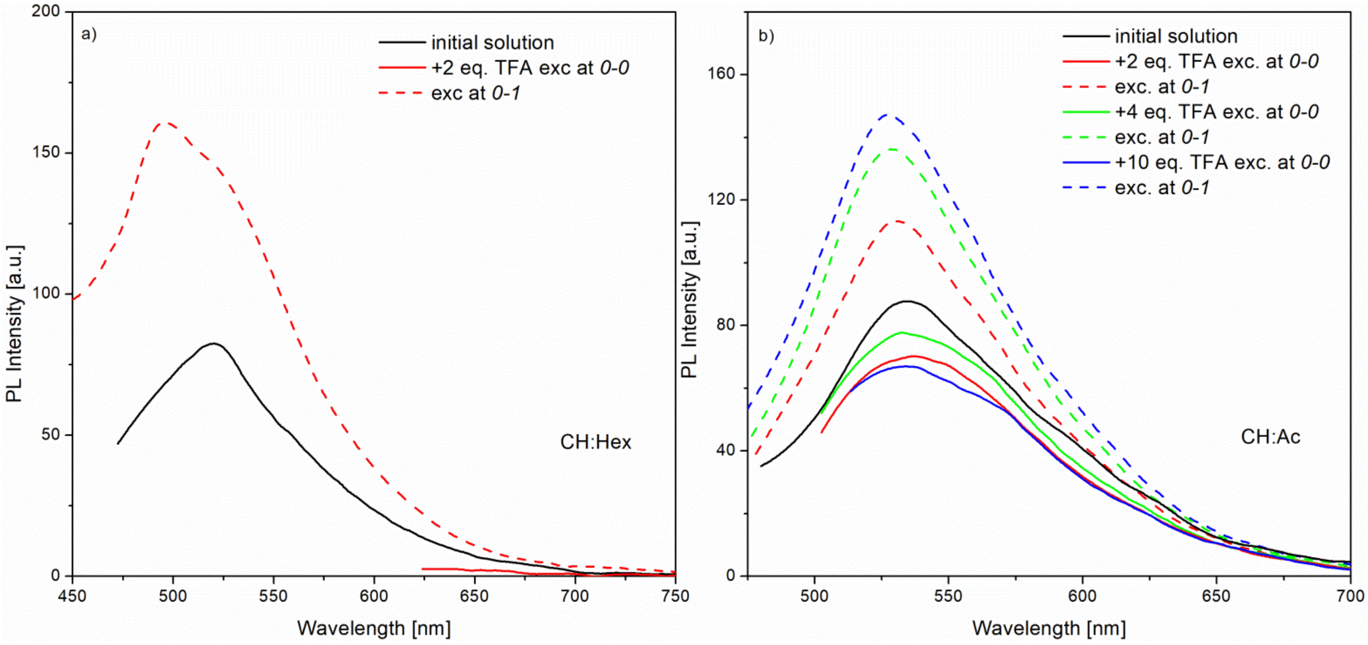

Figure S4. Photoluminescence spectra of **PAz1** solutions in chloroform:*n*-hexane (a) and chloroform:acetone (b), upon excitation with various wavelengths during sequential addition of dopant.

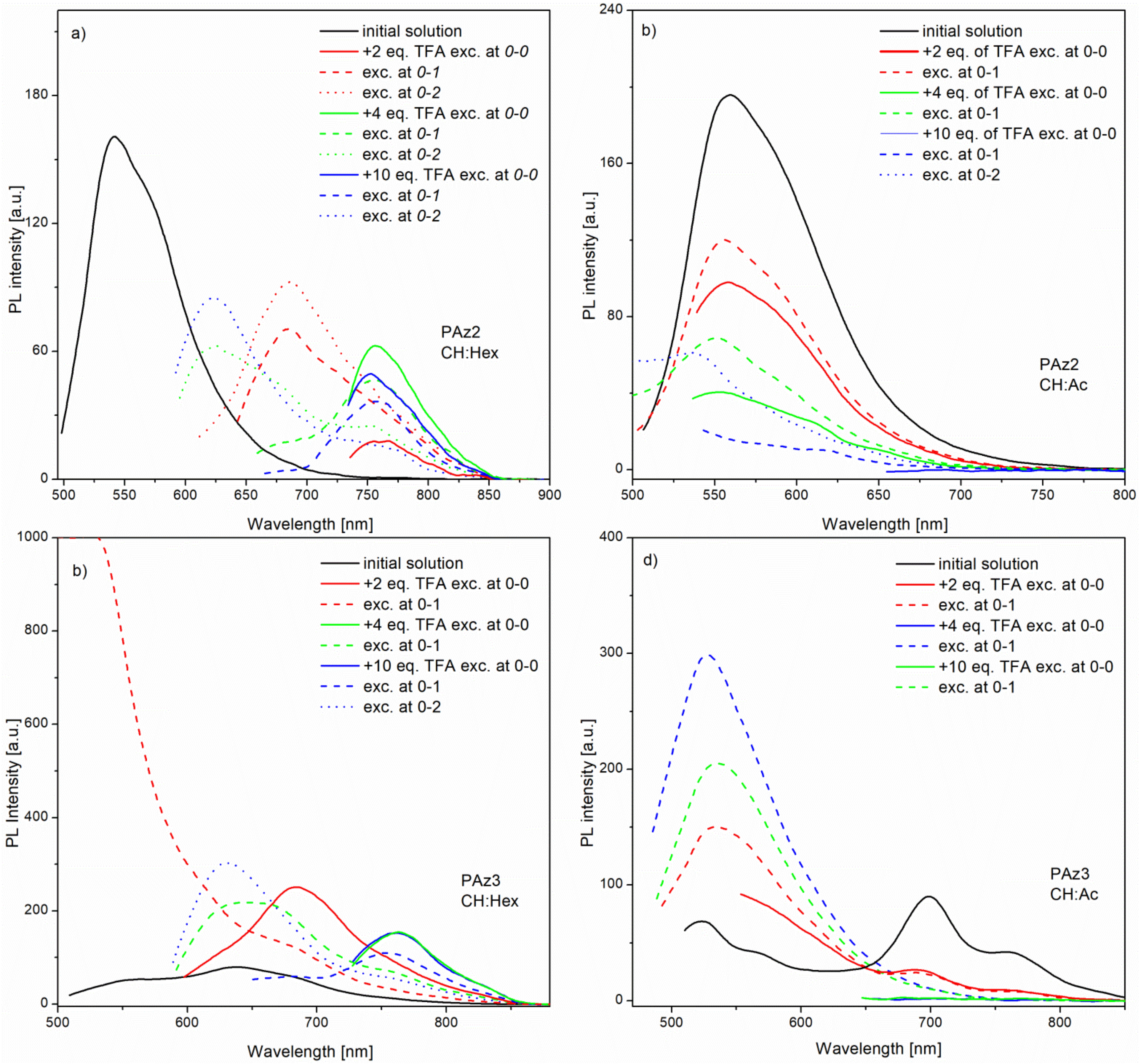

Figure S5. Photoluminescence spectra of **PAz2** (a, b) and **PAz3** (c, d) solutions in chloroform:*n*-hexane (a, c) and chloroform:acetone (b, d), upon excitation with various wavelengths during sequential addition of dopant
